# *Physalis angulata* induces *in vitro* differentiation of murine bone marrow cells into macrophages

**DOI:** 10.1186/1471-2121-15-37

**Published:** 2014-10-03

**Authors:** Bruno José Martins da Silva, Ana Paula D Rodrigues, Luis Henrique S Farias, Amanda Anastácia P Hage, Jose Luiz M Do Nascimento, Edilene O Silva

**Affiliations:** 1 Instituto de Ciências Biológicas, Laboratório de Parasitologia e Laboratório de Biologia Estrutural, Universidade Federal do Pará, Avenida Augusto Corrêa, 01, Bairro Guamá, 660975-110 Belém, Pará, Brazil; 2 Instituto Nacional de Ciência e Tecnologia em Biologia Estrutural e Bioimagem, Rio de Janeiro, Brazil; 3 Laboratório de Microscopia Eletrônica, Instituto Evandro Chagas, Secretaria de Vigilância em Saúde do Ministério da Saúde, Belém, Pará, Brazil; 4 Instituto de Ciências Biológicas, Laboratório de Neuroquímica Molecular e Celular, Universidade Federal do Pará, Avenida Augusto Corrêa, 01, Bairro Guamá, 660975-110 Belém, Pará, Brazil

**Keywords:** Cell differentiation, Bone marrow cells, *Physalis angulata*

## Abstract

**Background:**

The bone marrow is a hematopoietic tissue that, in the presence of cytokines and growth factors, generates all of the circulating blood cells. These cells are important for protecting the organism against pathogens and for establishing an effective immune response. Previous studies have shown immunomodulatory effects of different products isolated from plant extracts. This study aimed to evaluate the immunomodulatory properties of aqueous *Physalis angulata* (AEPa) extract on the differentiation of bone marrow cells.

**Results:**

Increased cellular area, higher spreading ability and several cytoplasmatic projections were observed in the treated cells, using optical microscopy, suggesting cell differentiation. Furthermore, AEPa did not promote the proliferation of lymphocytes and polymorphonuclear leukocytes, however promotes increased the number of macrophages in the culture. The ultrastructural analysis by Transmission Electron Microscopy of treated cells showed spreading ability, high number of cytoplasmatic projections and increase of autophagic vacuoles. Moreover, a high level of LC3b expression by treated cells was detected by flow cytometry, suggesting an autophagic process. Cell surface expression of F4/80 and CD11b also indicated that AEPa may stimulate differentiation of bone marrow cells mainly into macrophages. In addition, AEPa did not differentiate cells into dendritic cells, as assessed by CD11c analysis. Furthermore, no cytotoxic effects were observed in the cells treated with AEPa.

**Conclusion:**

Results demonstrate that AEPa promotes the differentiation of bone marrow cells, particularly into macrophages and may hold promise as an immunomodulating agent.

## Background

The hematopoietic tissue, bone marrow, is responsible for generating all circulating blood cells [1]. Hematopoietic stem cells undergo the process of maturation and differentiation in the presence of cytokines and growth factors present in the marrow microenvironment, giving rise to myeloid and lymphoid progenitor cells [2,3]. These myeloid progenitors, when stimulated, differentiate and give rise to blood cells, macrophages and dendritic cells (DCs), while the lymphoid lineage differentiates into T and B lymphocytes, natural killers (NK) cells and DCs [4,5].

The monocytes belong to the mononuclear phagocytic system and constitute about 3 to 8% of circulating leukocytes in the blood [6,7]. After three days in the circulating blood, monocytes begin the migration process to tissues where they differentiate into macrophages and DCs [6,7]. During the differentiation of monocytes into macrophages, several cellular changes are observed, such as increased cell size, increased number of organelles and the induction of the autophagic process [8,9]. Autophagy is essential for monocyte-macrophage differentiation; reports demonstrate that some monocytes cannot survive if the autophagy process is blocked and, if they are to survive, the differentiation process becomes defective inhibiting differentiation of cells into macrophages [9].

Macrophages express specific molecules on their surface, including the F4/80 and CD11b/MAC-1 proteins, which are markers of the differentiation process and allow macrophages to differentiate into other cell types [10,11]. These molecules are also involved in the process of cell adhesion and in the migration to sites of intracellular pathogen invasion [10]. Macrophages are important for maintaining an efficient innate immune response, having the ability to migrate to the site of invasion, recognizing the aggressor, phagocytosing and eliminating the pathogen [3,12].

In recent years, there has been a growing interest in the use of natural products to induce proliferation and differentiation of bone marrow cells [13-16]. In this context, *Physalis angulata* (Pa), which is a herbaceous plant, has been reported to possess several activities, among them, diuretic, antipyretic, analgesic [17], antinociceptive, anti-inflammatory and immunomodulatory [18,19] properties. Phytochemical studies of *P. angulata* demonstrate that extracts from this plant contains glucocorticoids, flavonoids, physalins (D, I, G, K, B, F, E), physagulins (E, F and G), and withanolides [20,21]. It is possible that the immunomodulatory effects of this plant may occur due to hematopoietic-supportive activities, through the activation of resident macrophages, which undergo several morphological changes, such as an increase in spreading and adhesion abilities, phagocytosis activity, ROS generation, antigen presentation and cytokine production. Therefore, the aim of this study was to evaluate the modulatory activity of AEPa on the cell differentiation process of monocyte-derived bone marrow cells in macrophages.

## Methods

### Preparation of the aqueous extract from roots of *Physalis angulata* (AEPa)

Roots of the *Physalis angulata* (Solanaceae) plant were collected in Pará state, Brazil. Roots were cut to produce the aqueous extract. AEPa was prepared as described by Bastos et al. [18]. The voucher specimen (no. 563) was deposited in the herbarium of the Emilio Goeldi Museum (Belém, Pará, Brazil). One mg/mL of aqueous extract from the root of *Physalis angulata* (AEPa) was dissolved in Dulbecco’s Modified Eagle’s Medium (DMEM) or RPMI and used as the standard solution for assays.

### Bone marrow cells isolation

Bone marrow cells (BMCs) were isolated from the femurs of male mice BALB/c (*Mus musculus*) aged 6–12 weeks. The animals were sacrificed in a CO_2_ chamber (Insight®) and the femurs were dissected under laminar flow and washed with sterile phosphate buffered saline (PBS). The epiphyses were then removed [22], and cells were homogenized and diluted in DMEM containing 10% FBS, maintained in 12, 24 or 96-well plates at 37°C in a 5% CO_2_ atmosphere. The experiments and study were carried out in accordance with current Brazilian animal protection laws (Lei Arouca number 11.794/08) in compliance with the National Council for the Control of Animal Experimentation (CONCEA, Brazil). The protocol was approved by the Committee on the Ethics of Animal Experiments of the Federal University of Pará (CEPAE/ICB/UFPA - grant number 086–12).

### Treatment of bone marrow cells

BMCs were cultured in the presence of 100 μg/mL of AEPa (1 mg/mL stock solution) for 24, 48, 72 and 96 hours. In some assays, BMCs were treated with 100 nM macrophage colony-stimulating factor (M-CSF), as positive control for differentiation. M-CSF and AEPa were added to the cultures every 24 hours until the end of each test, without replacing the culture medium.

### Cell viability tests

To assess the viability of the BMCs treated with AEPa, three tests were performed as described below.

#### Method Thiazolyl Blue (MTT)

MTT is a soluble salt, which is converted by mitochondrial dehydrogenases into formazan blue crystal. This assay is based on the mitochondrial-dependent reduction of 3-(4,5-dimethylthiazol-2-yl)-2,5-diphenyl tetrazolium bromide (MTT) to formazan. The procedure was performed according to Fotakis and Timbrell [23], with some modifications.

BMCs were cultured and treated with 25, 50 or 100 μg/mL AEPa for 24, 48, 72 and 96 hours. Subsequently, cells were incubated with 0.5 mg/mL MTT diluted in PBS and incubated at 37°C in a humidified atmosphere containing 5% CO_2_ for 3 hours. Two-hundred μL of DMSO were added to each well to solubilize formazan crystals and the plate was incubated under agitation for 10 minutes. The resulting solution was read in a microplate reader (BIO-RAD Model 450 Microplate Reader) and absorbance was recorded at an optical density (OD) of 570 nm. As a negative control, cells were killed with a 15% solution of formaldehyde in PBS.

#### Detection of the mitochondrial membrane potential (JC-1)

JC-1 is a fluorescent dye that measures the mitochondrial membrane potential (ΔΨ) of cells. The loss of this potential serves as an indicator of apoptosis, where this dye remains in its monomeric form and emits a green fluorescence. Living cells form the “J-aggregates” which emit a red fluorescence.

BMCs were treated with 100 μg/mL AEPA for 96 hours. Subsequently, cells were incubated with JC-1 (1 μM) for 30 min at 37°C. After incubation, the cells were washed and resuspended in PBS. Fluorescence data were obtained using a flow cytometer (BD FACSCantoII TM) at an excitation wavelength of 488 nm, where JC-1 monomers emit fluorescence at 529 nm and J-aggregates emit at 590 nm. A total of 10.000 events were acquired for each sample and the data were obtained by flow cytometer BD FACSCantoII. The data were analyzed using WinMDI version 2.9 (Joseph Trotter) software. The gate was determined using unstained BMCs controls (Additional file 1). The data were analyzed using WinMDI version 2.9 (Joseph Trotter) software.

### Detection of apoptosis and necrosis of BMCs treated with AEPa

For detection of apoptosis and necrosis of BMCs treated with AEPA, Annexin V-FITC (Invitrogen) and PI (Sigma) were used, respectively. BMCs were treated with 100 μg/mL AEPa and cultured for 96 hours. After treatment, these cells were incubated for 30 minutes with 10 μg/mL Annexin V-FITC and then incubated with 25 μg/mL PI for 30 minutes. Finally, the cells were washed with PBS and data obtained by flow cytometry. A total of 10.000 events were acquired for each sample in the region that corresponded to the BMCs and the gates were determined using unstained controls (Additional file 1).

### Light microscopy (LM)

BMCs were cultured and treated for 24, 48, 72 and 96 hours before dividing into three groups, control (non treated cells), treated with AEPa and M-CSF. Cells were fixed in a solution containing 3% paraformaldehyde in PHEM buffer (5 mM magnesium chloride, 70 mM potassium chloride, 10 mM EGTA, 20 mM HEPES, 60 mM PIPES), 0.1 M pH 7.2, stained with Giemsa and covered with Entellan® (Merck). Two hundred cells were counted per coverslip. Differentiated cell types such as lymphocytes, mononuclear phagocytes (monocytes and macrophages) and polymorphonuclear (PMN) were identified according to their morphological characteristics. Cells were counted and analyzed using an Olympus BX41 microscope.

### Morphometric analysis

The cytoplasmic area of the control group and treated BMCs (100 μg/mL of AEPa for 96 hours) was analyzed using the program Image J (NHI) software and images were obtained by light microscopy. This analysis was performed as described by Sokol et al. [24].

### Transmission Electron Microscopy (TEM)

Control and treated BMCs were fixed with 2.5% glutaraldehyde and 4% paraformaldehyde in 0.1 M sodium cacodylate buffer, pH7.2. The cells were washed in the same buffer and incubated in 1% osmium tetroxide and 0.8% potassium ferricyanide for 1 hour. The cells were dehydrated in graded acetone (50%, 70%, 90% and 2× 100%) and embedded in Epon resin (2:1, 1:1 and 1:2 - 100% acetone: Epon). Thin sections were contrasted with 5% uranyl acetate and lead citrate and finally observed with a LEO 906 E Transmission Electron Microscope.

### Detection of LC3b protein by flow cytometry

Treated and untreated BMCs were fixed with 3% paraformaldehyde and 0.1 M PHEM buffer, pH 7.2, for 30 minutes. Subsequently, cells were permeabilized with 0.1% Triton X-100, washed in PBS and incubated with 50 mM NH_4_Cl in PBS for 40 minutes.

The cells were incubated with polyclonal anti-LC3b antibody (Invitrogen Molecular Probes®) diluted 1:1000 in PBS with 1% BSA for 1 hour, then washed in PBS and incubated with a fluorescent secondary antibody (Alexa Fluor 488-labelled goat anti-rabbit IgG; Molecular Probes Invitrogen®) diluted 1:100 in PBS for 30 minutes. Data were obtained by flow cytometry (BD FACSCantoII) at an excitation wavelength of 488 nm. The results were analyzed by WinMDI version 2.9 (Joseph Trotter). For induction of autophagy, BMCs were cultured for 96 hours, washed with PBS and incubated for 3 hours with phosphate buffer, pH 7.2, at 37°C in 5% CO_2_ and used as a positive control for the autophagic process.

### Detection of cell surface markers by flow cytometry

Treated and untreated BMCs were fixed with 3% paraformaldehyde and 0.1 M PHEM buffer, pH 7.2, for 30 minutes. Cells were washed in PBS, pH 8.0, and incubated with 50 mM NH_4_Cl in PBS for 40 minutes. Next, the cells were incubated for 1 hour with anti-CD11c monoclonal antibody (DCs marker), anti-CD11b (Mac-1) and anti- F4/80 monoclonal antibody (mononuclear cells and macrophage markers, respectively), diluted 1:50 in PBS. Subsequently, cells were incubated with fluorescent secondary antibody conjugated with PE-goat anti-rat IgG, diluted 1:50 in PBS for 40 minutes. A positive control was treated with M-CSF (100 mM) and also maintained in parallel. All experiments were performed at least three times with treated and untreated cells. Data were obtained by flow cytometry (BD FACSCantoII) at an excitation wavelength of 546 nm and analyzed by WinMDI version 2.9 (Joseph Trotter) software.

### Statistical Analysis

All experiments were performed in triplicate and the results were analyzed by GraphPad Prism 5 (GraphPad Software, La Jolla, CA, USA). The means and S.D. of at least three experiments were determined. Analysis of variance (ANOVA) and Student’s t-test were used to compare data. The Tukey test was applied when necessary. All p-values <0.05 were considered as statistically significant.

## Results

### Effect of AEPa on BMCs cell viability

**Figure 1:**
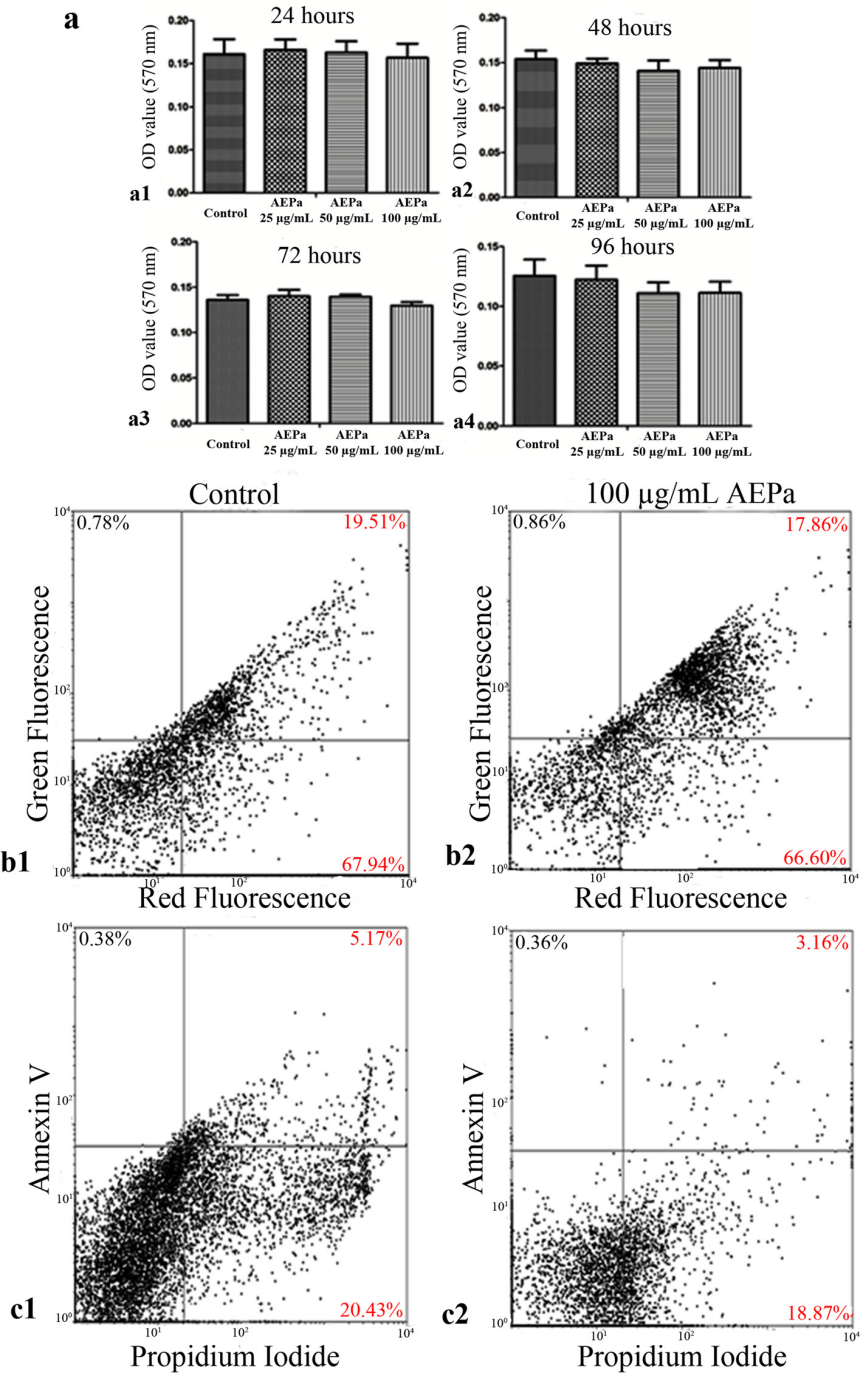
**Cellular viability of bone marrow cells (BMCs) treated with AEPa, as measured by MTT, JC-1, propidium iodide and annexin V assays. a)** Cell viability was determined using the MTT assay. Treatment of BMCs maintained in culture after 24, 48, 72 and 96 hours with different concentrations of AEPa, (25, 50 and 100 μg/mL). Data are expressed as mean ± SD of three independent experiments. ANOVA followed by Tukey test, p <0.05. **b1-b2)** Determination of mitochondrial membrane potential (Δψ_m)_ by flow cytometry. Δψ_m_ detected by JC-1 in untreated BMCs **(b1)** or treated cells with 100 μg/mL of AEPa after 96 hours **(b2)** (results from 10.000 events analyzed). **c1** and **c2)** Determination of apoptosis and/or necrosis analyzed by flow cytometry. **c1)** Untreated cells. **c2)** BMCs treated with 100 μg/mL AEPa and maintained in culture after 96 hours. The percentages of apoptotic and necrotic cells are presented in the dot plots and are representative of one of three independent experiments (results from 10.000 events analyzed).

BMCs were treated with 25, 50 and 100 μg/mL AEPa for 24-96 h and cell viability analyzed by the MTT assay (Figure 1a). Alternatively, BMCs were treated with 100 μg/mL for 96 hours and loaded with JC-1 (Figure 1b1 and 1b2) or stained with PI and Annexin-V (Figure 1 c1 and 1 c2). No cytotoxic effect of AEPa was observed in cells treated for 24, 48, 72 and 96 hours, when compared to the control group, as shown by the MTT assay. Labeling with JC-1 and PI and annexin-V demonstrated that treated cells remain viable following 96 hours of culture.

### Quantitative analysis of adherent cells

To evaluate the effect of AEPa on the BMCs, a quantitative analysis was performed and identified the following cell types, including lymphocytes, PMN and mononuclear phagocytes.

#### Lymphocytes

**Figure 2:**
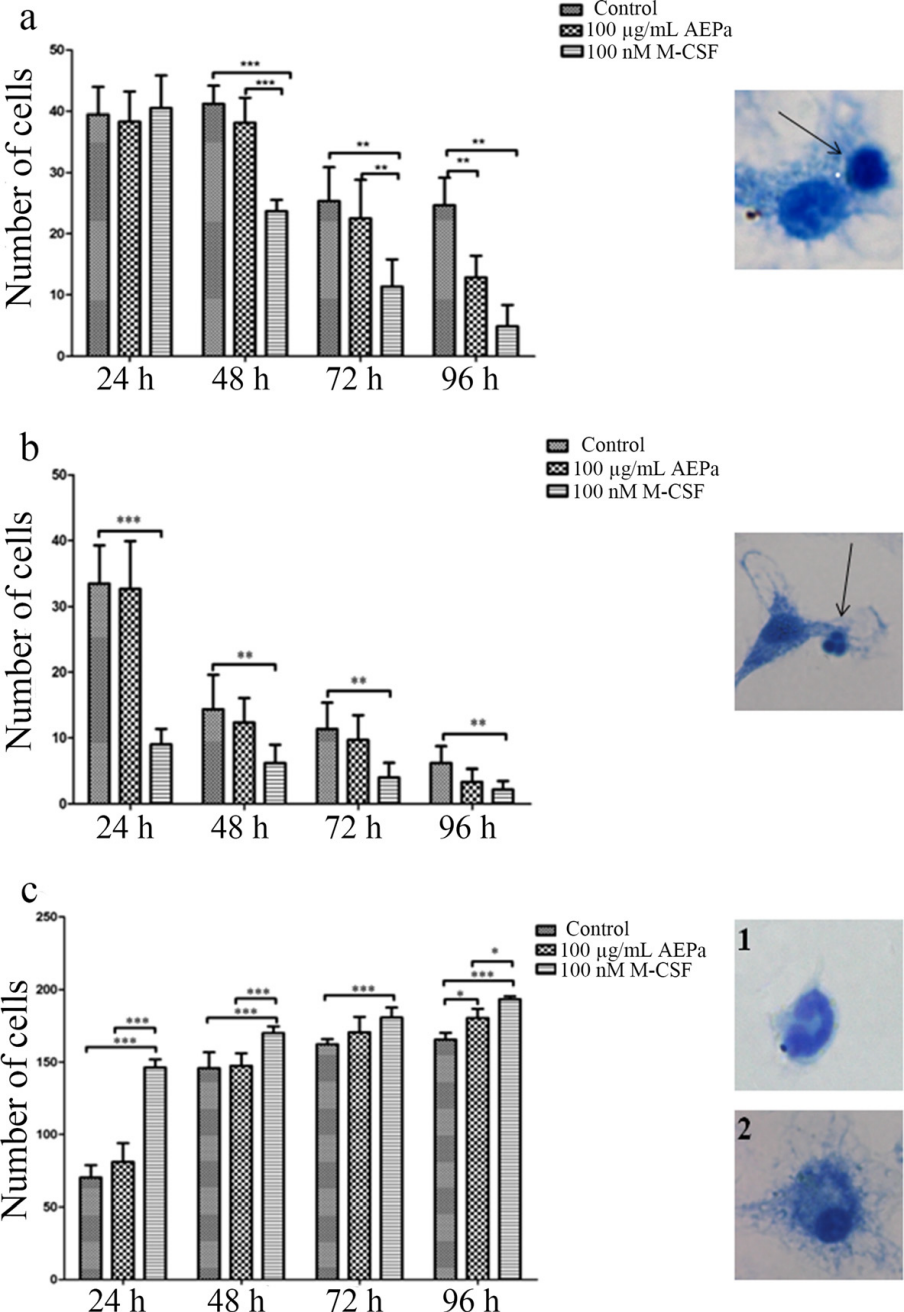
**Differential cell count in BMCs cultures after 24, 48, 72 and 96 hours.** Cells were treated with 100 μg/mL of AEPa, 100 nM of M-CSF and compared to the control group. **a)** Lymphocytes. Inset, lymphocytes (arrow), cells with a small cytoplasmic area and large nucleus. **b)** Polymorphonuclear. Inset, showing polymorphonuclear (arrow), cells with two or more nuclei arranged in the cytoplasm. **c)** Mononuclear phagocytes. Inset 1, showing monocyte, cell with a small cytoplasmic area and nucleus in a horseshoe shape and, inset 2 showing macrophage, cell with a large nucleus and evident cytoplasm. The values are expressed as means ± SD and compared with the control group. ANOVA, followed by Tukey test, p <0.05.

Lymphocytes were characterized as cells with a small cytoplasmic area and large nucleus. From 96 hour of treatment, a reduction of 40% ± 8 in the number of lymphocytes was observed when compared with untreated cells (Figure 2a).

#### PMN cells

PMN cells have two or more nuclei arranged in the cytoplasm. No significant difference was observed between the control group and the group treated with AEPa for 96 hours (Figure 2b).

#### Mononuclear cells

Mononuclear cells constitute monocytes (cells with a small cytoplasmic area and nucleus in a horse-shoe shape) and macrophages (large nucleus and evident cytoplasm). A significant increase in the number of cells with macrophage characteristics was observed in the cultures treated with AEPa (8% ± 3) for 96 hours, when compared to the control group (Figure 2c).

### AEPa induces morphological alterations and increases cellular area in BMCs

**Figure 3:**
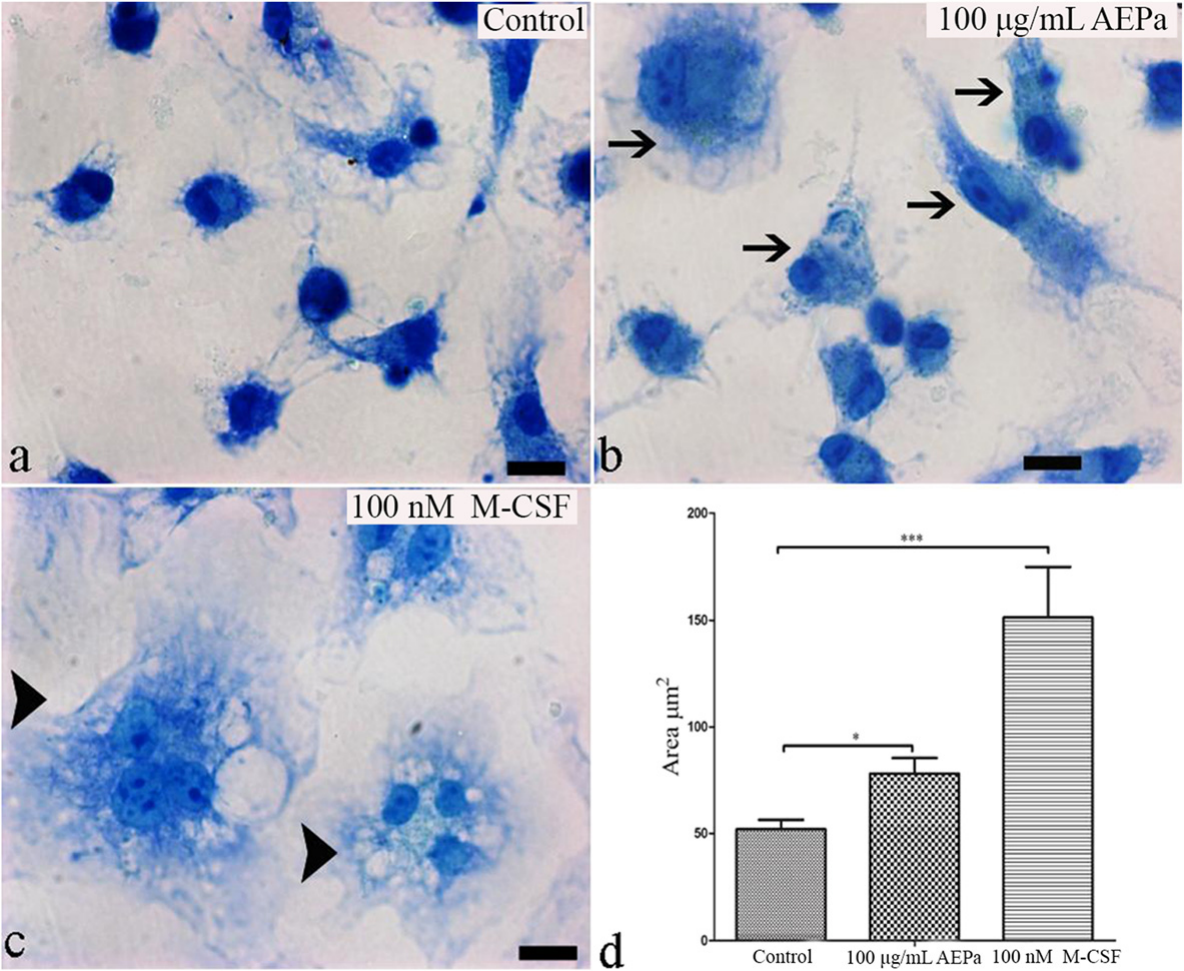
**Cytological evaluation of BMCs using Giemsa stain.** Treated cells were incubated for 96 hours. **a)** Untreated cells. **b)** Cells treated with 100 μg/mL AEPa, note the increased cell spreading, cytoplasmic volume and cells with characteristics of activated macrophages (arrow). **c)** Cells treated with 100 nM of M-CSF. Cells after treatment with M-CSF presented a significant spreading ability, increased cellular area and most of the cells are undergoing cell fusion processes (head arrows). Scale bar 10 μm. **d)** Morphometric analysis showed increased cell area for cells treated with AEPa or M-CSF, compared with control cells. Data are presented as means ± SD, p < 0.05.

**Figure 4:**
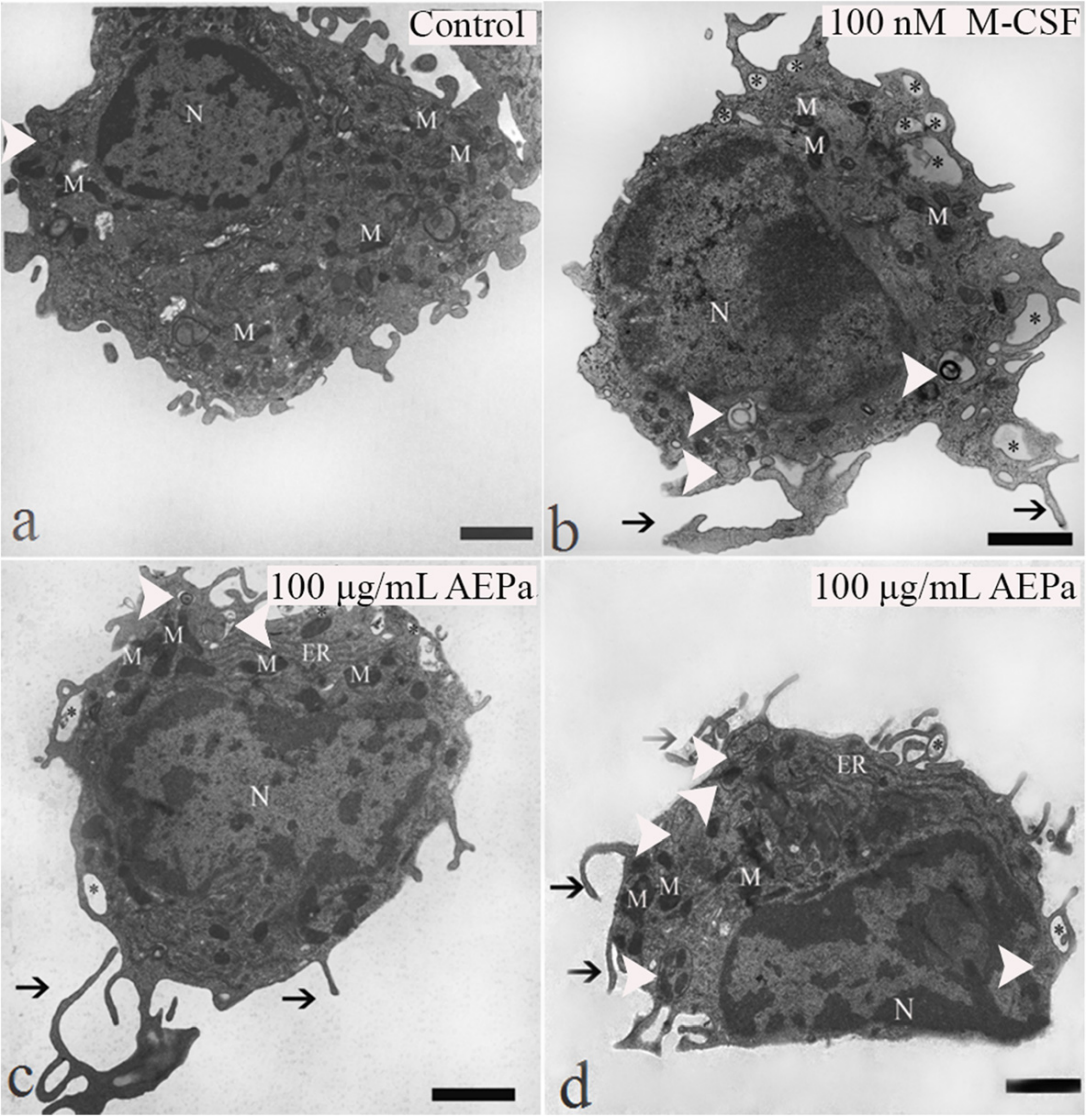
**Ultrastructural analysis of BMCs.** Treated cells were incubated for 96 hours. **a)** Untreated control. **b)** Cells treated with 100 nM M-CSF. **c**, **d)** Cells treated with 100 μg/mL of AEPa. Cells treated with M-CSF and AEPa presented filopodia (arrows), cytoplasmic vacuoles (*), mitochondria and abundant endoplasmic reticulae and presence of autophagic vacuoles (head arrows). Bars: 5 μm. N: Nucleus, M: Mitochondria, ER: Endoplasmic reticulum.

**Figure 5:**
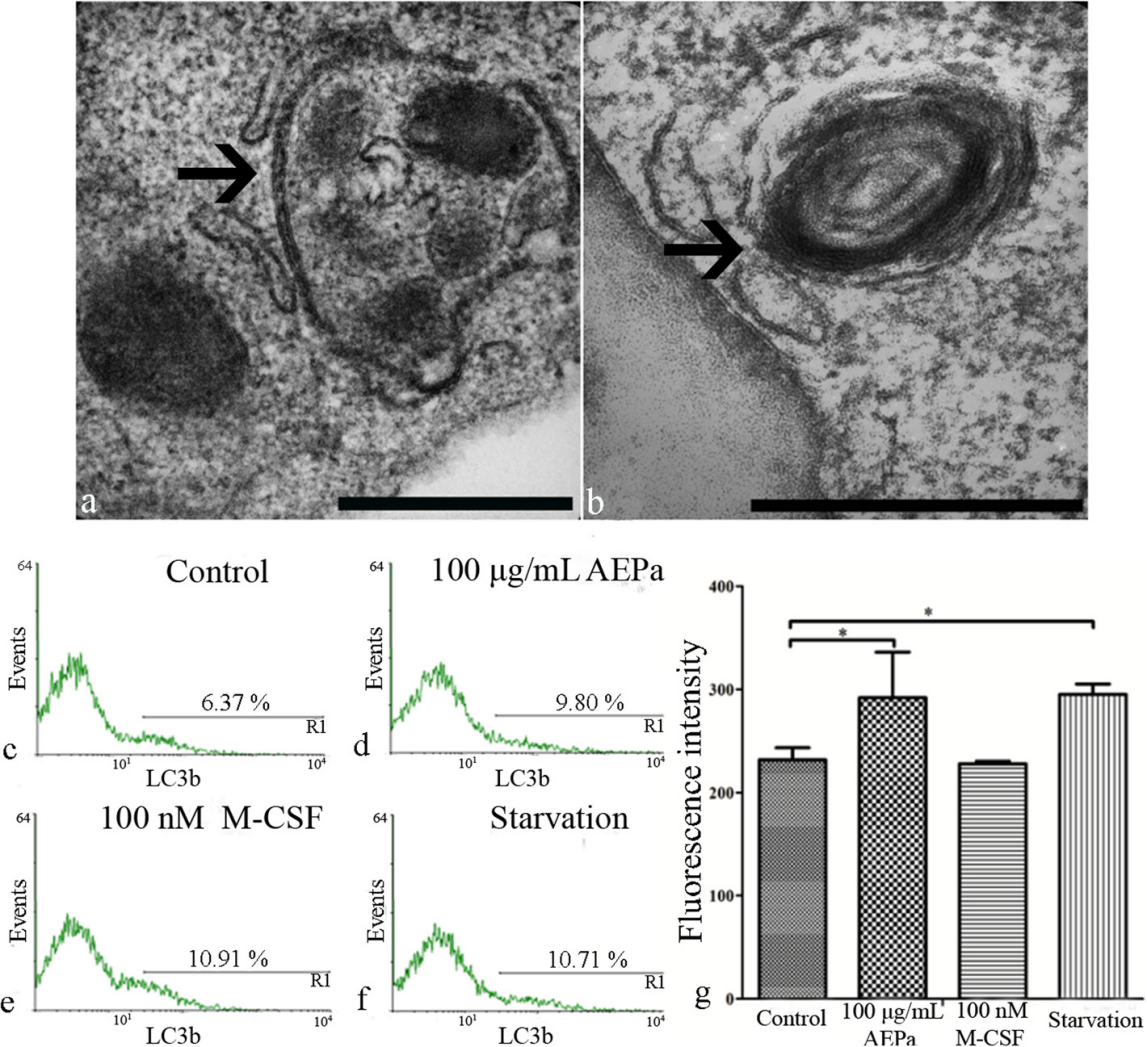
**Detection of autophagic process in BMCs.** Treated cells were incubated for 96 hours. **a-b)** Ultrastructural analysis of BMCs treated with AEPa. Cells treated with AEPa presented autophagic vacuoles in the cytoplasm (arrows). Bars: 0.5 μm. **c)** Untreated control. **d)** Cells treated with 100 μg/mL AEPa. **e)** Cells treated with 100 nM M-CSF. **f)** Starvation. **g)** Fluorescence intensity of BMCs stained with LC3b. ANOVA followed by Tukey test, p <0.05.

Control and BMCs treated with AEPa were analyzed by LM and TEM. Morphological alterations were observed in 100 μg/mL AEPa-treated cells that were characteristic of activated cells. An increase in cytoplasmic area, spreading ability and a high number of cytoplasmatic projections were also observed (Figure 3b). Morphometric analysis showed significant increase in the area occupied by cytoplasm in cells treated with AEPa, when compared to the control group (Figure 3d).To investigate possible ultrastructural changes in cells treated with AEPa, TEM was performed. BMCs treated with AEPa presented nuclei with abundant euchromatin, an apparently increased number of endoplasmic reticuli (ER), numerous mitochondria, which are characteristic of intense cell metabolism, and numerous cellular projections (Figure 4c and d). The presence of cytoplasmic vacuoles and structures suggestive of autophagic vacuoles were observed in the cytoplasm of AEPa-treated cells (Figure 5a and b).

### Induction of autophagy in BMCs

To test whether AEPa induces autophagy in BMCs, cells were treated for 96 hours and the expression of LC3b evaluated by flow cytometry. LC3b is a specific marker for autophagy in mammalian cells; treated BMCs presented higher fluorescence intensity when compared to the untreated control group. The staining of cells treated with AEPa was similar to that of the control group after starvation and to that of the group of cells treated with M-CSF (Figures 5c-g).

### Detection of cell surface markers by flow cytometry

**Figure 6:**
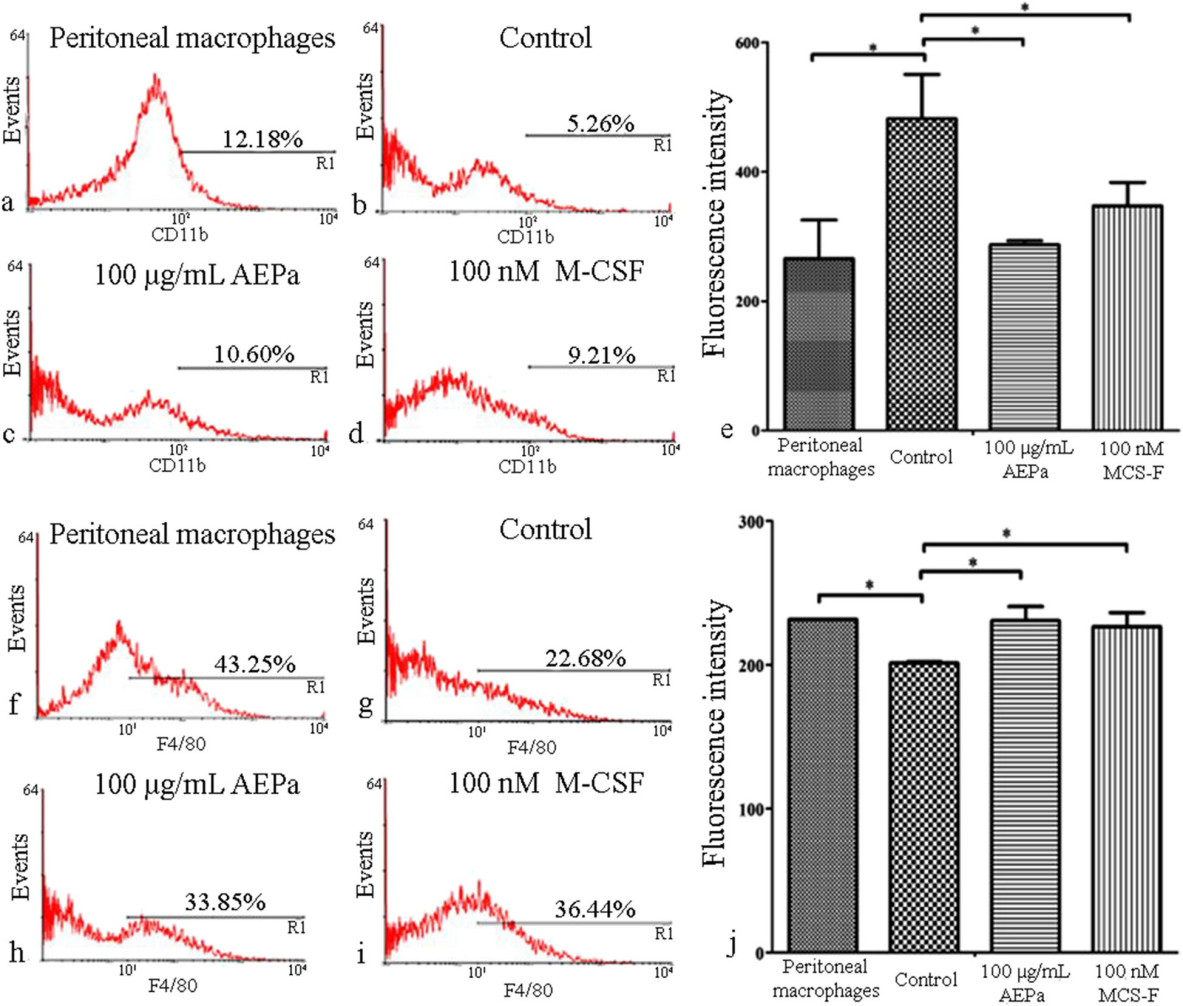
**Expression of CD11b and F4/80 on BMCs.** Treated cells were incubated for 96 hours. **a**-**d)** Detection of the CD11b surface marker by flow cytometry. **e)** Fluorescence intensity of BMCs labeled with CD11b. **f**-**i)** Flow cytometric analysis of F4/80 surface marker. **j)** Fluorescence intensity of BMCs stained with F4/80. ANOVA was followed by Tukey test, p <0.05.

To determine whether AEPa promotes the differentiation of BMCs into macrophages, the expressions of the surface proteins F4/80, CD11b and CD11c were assessed on BMCs by flow cytometry. An increased of expression of CD11b (Figure 6c) and F4/80 (Figure 6h and 6j) and were observed on AEPa-treated cells. The same expression levels were observed in the positive-control groups, consisting of peritoneal macrophages (Figure 6a, for CD11b and 6f for F4/80) and BMCs stimulated with M-CSF (Figure 6d for CD11b and 6i for F4/80), in comparison with untreated cells. Analysis of the fluorescence intensity showed that there was a decreased staining of CD11b protein in AEPa-treated cells as also observed in the group treated with M-CSF and peritoneal macrophages (Figure 6e). Furthermore, CD11c labeling showed no significant difference in levels expression compared with untreated cells, AEPa treated cells or M-CSF group (Additional file 2), showing that AEPa and M-CSF does not stimulate the differentiation of BMCs into dendritic cells.

## Discussion

A great number of herbal products have been used in folk medicine due to their immunomodulatory actions [15,16,25,26]. Extracts and physalins obtained from *P. angulata* exhibit diverse biological properties, including, analgesic, anti-inflammatory and immunomodulatory activities [18,19,27-29]. AEPa exhibits beneficial effects on carragenin-induced air pouch inflammation through its immunomodulatory action [19]; however, the direct action of AEPa on bone marrow remains unknown. Here, we demonstrate for the first time that AEPa has an immunomodulatory effect on BMCs, differentiating cells into macrophages. Chemical analyses from our group have found that aqueous extracts of the dried root of *P. angulata* contain physalins D, E, F and G (unpublished data). We hypothesize that the immunomodulatory effects of AEPa may derive from the presence of these physalins.

The differentiation of monocytes into macrophages or DCs in culture is most commonly achieved during 5 days, although a process of rapid differentiation within several hours can occur, depending on the stimulus used [30]. These interesting effects indicate that bone marrow-derived monocytes differentiate into macrophages; however, not all cell types respond in this same manner during AEPa treatment.

A quantification experiment was performed to identify the presence of different cell types in these cultures. Lymphocyte numbers were found to be significantly reduced in BMCs treated with AEPa for 96 hours; as such, AEPa does not stimulate the adhesion and proliferation of this cell type. Bastos et al. [19] showed that AEPa had an inhibitory effect on lymphocyte proliferation, particularly on T cells. These results are in agreement with those observed by Yu et al. [31], who demonstrated that physalin H obtained from *P. angulata* presents an immunosuppressive activity, thus preventing the proliferation of T cells.

BMCs treated with AEPa showed a significant increase of mononuclear cells when compared to control. Morphological LM analysis showed that AEPa-treated cells had a higher spread ability and morphometric analysis revealed that treated cells showed an increase in cellular area. In addition, TEM demonstrated that BMCs, treated for 96 hours with 100 μg/mL AEPa, presented nuclei with abundant euchromatin, augmented ER, elongated mitochondria and the presence of numerous cellular projections. Several changes are observed during the process of differentiation of monocytes into macrophages such as altered expression of surface proteins, increased cell size, increased number of organelles and autophagic induction [8,9].

Another characteristic feature seen in AEPA- and M-CSF-treated BMCs was the presence of autophagic vacuoles in the cytoplasm, which was not observed in untreated cells. Analysis by flow cytometry revealed a significant increase in LC3b protein on treated cells, when compared to the control group, indicating an autophagy process. Autophagy is very important during the process of monocyte differentiation into macrophages, allowing the proliferation of these cells and preventing death by apoptosis [9]. The autophagic activation mechanism can be induced by various routes, one of which is by ER stress, and the response of the organism to autophagy can be varied, allowing cell survival or leading to non-apoptotic death [32,33]. Our results show that AEPa-treated cells presented autophagic characteristics and this mechanism was not deleterious to the cells, since there was no reduction in cell viability. Thus, we may suggest that autophagy favors cell survival, allowing the differentiation of monocytes into macrophages.

Macrophages may elicit an altered pattern of surface markers, as compared to non-differentiated cells. The surface proteins, CD11b and F4/80, are examples of molecules that change after macrophage differentiation [34]. CD11b can be found on the surface of neutrophils, DCs, lymphocytes and macrophages, but is highly expressed on the monocyte surface [10]. However, the F4/80 molecule is expressed only on the macrophage surface [11]. Flow cytometry demonstrated a significant decrease in CD11b levels on AEPa-treated cells when compared to the control group. Results indicate that monocytes were the most common cell type in the untreated BMCs, because this group had a high staining for the CD11b protein. On the other hand, cells found in the cultures treated with AEPa or M-CSF were mainly macrophages as demonstrated by the decrease in CD11b protein expression. To confirm that macrophages were the major cell type in the culture population, expression of the macrophage-specific protein, F4/80, was determined. There was a significant increase in surface F4/80 on the AEPA-treated cells, where levels were similar to those observed in the positive control (peritoneal macrophages) and the M-CSF-treated group. These results suggest that AEPa potentiates the differentiation of monocytes into macrophages.

Another important immune cell type that is derived from the monocyte lineage is the DCs; these are antigen-presenting cells (APCs) that play a major role in bridging of the innate and adaptative immune responses. When monocytes migrate to tissues they can differentiate into macrophages or DCs [3]. In this study, we have used the surface marker, CD11c, to determine the presence of this cell type. No significant difference in the expression levels for CD11c on AEPa-treated BMCs was observed. Thus, AEPa was not able to stimulate the differentiation and maturation of bone marrow mononuclear cells in DCs. It is important to emphasize that no cytotoxic effects were observed in the cells treated with AEPa. The search for new products, without cytotoxic effects on mammalian cells, is important for the development of agents that can induce the differentiation and/or maturation of BMCs into macrophages. Since these cells participate in the initiation of the innate immune response, such agents may be able to promote the protection against several intracellular pathogens [12,26].

## Conclusion

AEPa seems to act on different aspects of cellular differentiation, with potential to act as an immunomodulatory agent, inducing the differentiation of BMCs into macrophages, which are important cells in the defense against pathogens.

## Abbreviations

BMCs: Bone marrow cells; DMEM: Dulbecco’s modified Eagle’s medium; FBS: Fetal bovine serum; AEPa: Aqueous extract from root of *Physalis angulata*; M-CSF: Macrophage colony-stimulating factor; MTT: 3-(4,5-dimethylthiazol-2-yl)-2,5-diphenyl tetrazolium bromide; PI: Propidium iodide; LM: Light microscopy; TEM: Transmission electron microscopy; BSA: Bovine serum albumine; DMSO: Dimethylsufoxide.

## Competing interests

The authors declare that they have no competing interests.

## Authors’ contributions

BJMS performed all experiments and wrote the manuscript. LHSF and AAPH performed flow cytometry analysis. APDR performed the LM and TEM analyzes. BJMS, APDR, EOS and JLMN were involved in the discussion of results and manuscript editing. All authors read and approved the final manuscript.

## Supplementary Material

Additional file 1 Flow citometry of unstained BMCs controls elucidating gates for further analysis of treated cells. Click here for file

Additional file 2 **Detection of the CD11c surface marker by flow cytometry on BMCs.** Treated cells were incubated for 96 hours. **a)** Untreated control. **b)** Cells treated with 100 μg/mL AEPa. **c)** Cells treated with 100 nM M-CSF. **d)** Fluorescence intensity of BMCs labeled with CD11c. ANOVA followed by Tukey test. p <0.05. Click here for file
