# Supplementary figures and images for: Physalis angulata induces in vitro differentiation of murine bone marrow cells into macrophages
Source: BMC Cell Biol. 2014 Oct 3;15:37. doi: 10.1186/1471-2121-15-37 (PMC4665300; doi:10.1186/1471-2121-15-37)

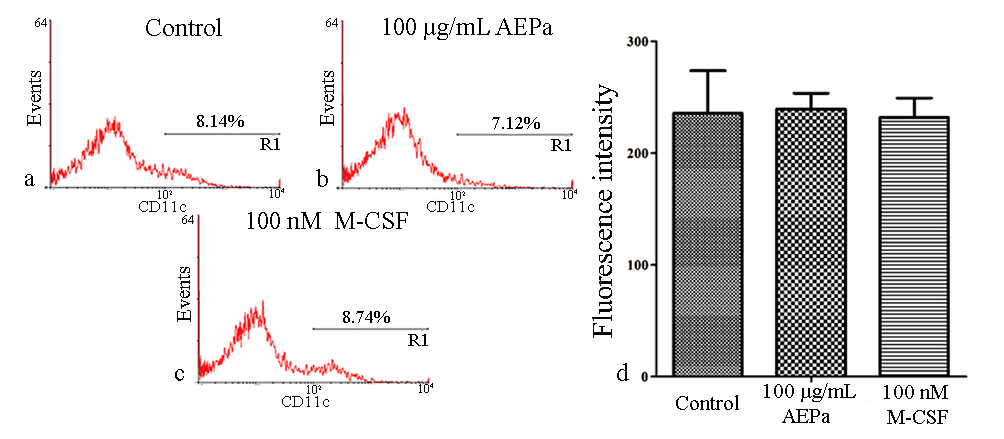

Supplement: Additional file 1 — Flow citometry of unstained BMCs controls elucidating gates for further analysis of treated cells. [file 1471-2121-15-37-S1.tiff]

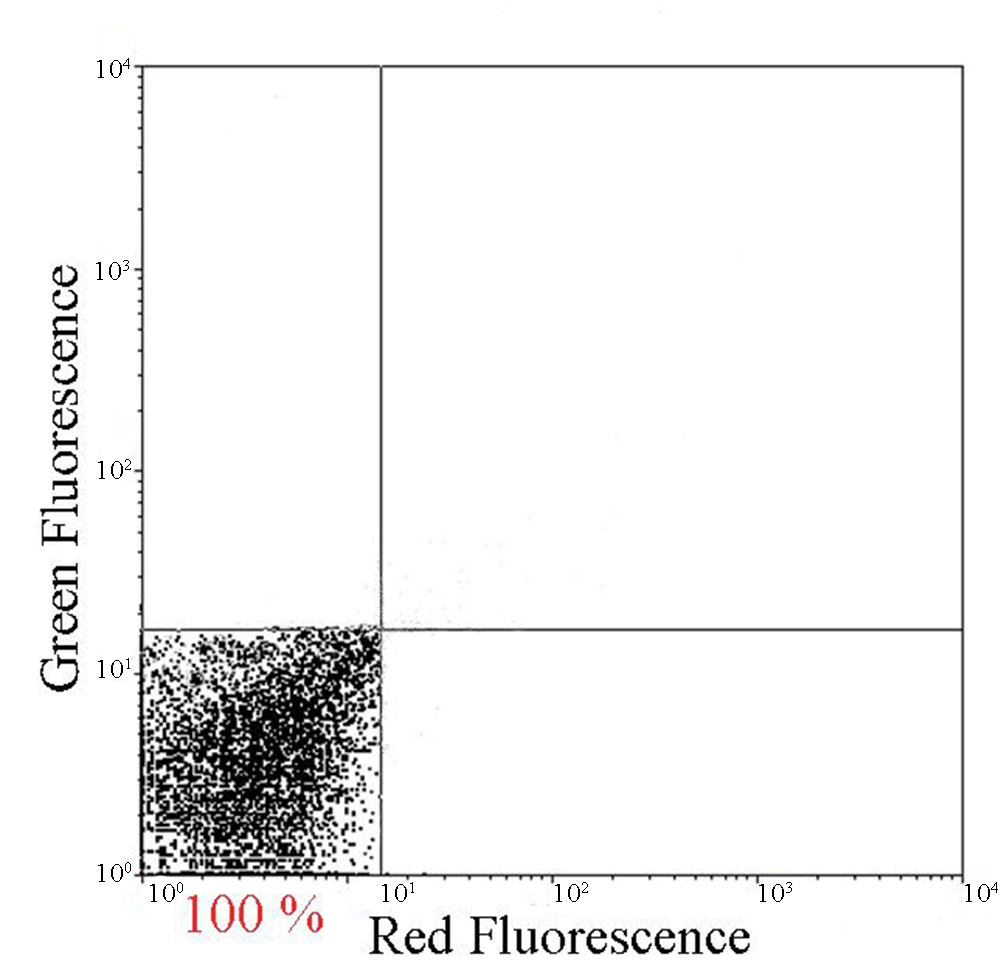

Supplement: Additional file 2 — Detection of the CD11c surface marker by flow cytometry on BMCs. Treated cells were incubated for 96 hours. a) Untreated control. b) Cells treated with 100 μg/mL AEPa. c) Cells treated with 100 nM M-CSF. d) Fluorescence intensity of BMCs labeled with CD11c. ANOVA followed by Tukey test. p <0.05. [file 1471-2121-15-37-S2.tiff]
